# Indian research on aging and dementia

**DOI:** 10.4103/0019-5545.69227

**Published:** 2010-01

**Authors:** K. S. Shaji, V. P. Jithu, K. S. Jyothi

**Affiliations:** Department of Psychiatry, Medical College, Thrissur - 680 596, Kerala, India

**Keywords:** Aging, mental health, dementia, late onset depression

## Abstract

All the articles published in the Indian Journal of Psychiatry (IJP) from 1958 to 2009 on aging, dementia and other mental health issues of late life were systematically reviewed. There were only a limited number of research articles on dementia in the IJP. Most of the Indian studies on dementia were published elsewhere. People above the age of 60 years constitute about 5% of patients seen in tertiary care settings. High prevalence of psychiatric morbidity was reported among community resident older people. Depression was the commonest mental health problem in late life. We need to develop community-based interventions for management of common conditions like depression in late life. The effectiveness of these interventions needs to be established. It is important to identify risk factors for depression and dementia in our population. We could then try and modify these factors to reduce the prevalence of these conditions.

## INTRODUCTION

India is going through a phase of rapid demographic aging. The number of people with dementia and other late life mental health problems are expected to increase in the near future. Research and dissemination of research findings are important for service development and training. This paper aims to review the published research on people above the age of 60 years. The focus of the review was only on the articles published in Indian Journal of Psychiatry (IJP). Indian studies published elsewhere will be referred to in the discussion.

## MATERIALS AND METHODS

An electronic search was done to identify the articles available on the IJP website. All the issues of the journal from 1958 to the current issue in 2009 were searched. The words ‘aging’; ‘aged’; ‘ dementia’; ‘elderly’; ‘geriatric’; ‘late onset’ ‘older people’ and ‘mild cognitive’ were used to generate the potential list of articles. These articles were assessed for relevance by seeing the abstract or full text. To be included in the review, the content of the article should have addressed issues related to aging, dementia or any mental health problem in late life. The selected articles were then reviewed in detail and the findings were summarized. The articles were broadly classified as research reports, editorials and other articles. Dementia and other cognitive disorders were considered together. Other late life mental health conditions were reviewed separately.

## RESULTS

There were nine research reports[1–8] and two case reports[9,10] on dementia. One of these was a study conducted in Sri Lanka.[8] Highlights of eight Indian studies are summarized in Table 1. There were five other studies which looked at cognitive disturbances due to other causes. Two reports from a study on delirium examined the prevalence of delirium in elderly medical patients and the risk factors.[11,12] Another study[13] looked at cognitive decline among older people admitted to the medical and surgical wards of a general hospital. Two other studies looked at the efficacy of herbal formulations in age-associated cognitive decline.[14,15]

**Table 1:** Dementia research (IJP: 1958-2009)

Study (location)	Setting	Subjects	Dementia diagnosis	Remarks
Somasundaram and Sarada Menon (1975) (Chennai, Tamil Nadu)	Psychiatric hospital	12 (inpatients)	Clinical	Neuropathological evaluation after cerebral biopsy
Kalyanasundaram *et al*. (1979) (Bengaluru, Karnataka)	Psychiatry and neurology hospital	40 (inpatients)	Clinical	Correlation of dementia severity and clinical, EEG and PEG findings
Khandelwal *et al*.(1992) (New Delhi)	Tertiary care	30 outpatients from neurosciences centre	DSM III -R	Describes behavioral symptoms in dementia
Kar *et al*. (2000) (Manipal, Karnataka)	Tertiary care	39 (inpatients)	ICD-10	Analysis of case records to see the differential diagnosis of various dementias
Shaji *et al*. (2009) (Ernakulam, Kerala)	Dementia care services	40	DSM IV criteria for dementia of Alzheimer’s type	Report nature and prevalence of behavioral symptoms
Shaji *et al*.(2009) (Thrissur, Kerala)	Tertiary care dementia clinic	137 (outpatients)	DSM IV and other criteria	Describes the weekly dementia clinic in a general hospital
Shaji *et al*.(2009) (Thrissur, Kerala)	Community	29 (community resident)	DSM IV	High prevalence of behavioral symptoms and caregiver burden
Prasad *et al*. (2009) (Bengaluru, Karnataka)	Tertiary care	51 (outpatient)	ICD-10	Retrospective analysis of case records. Audit of prescribed medication

There was one review article[16] and one article in the Continuing Medical Education (CME) section in the January-March issue of the journal[17] on dementia. The dementia supplement was published along with the January-March 2009 issue of the journal. It featured 15 invited articles[18–32] and two editorials[33,34] on dementia. Invited articles covered various aspects of dementia and were written by experts with special interest in dementia. Of the 35 publications on dementia and related disorders, 30 were published in the last ten years. The year 2009 alone saw 23 papers on dementia, most of them in the dementia supplement.

We found 35 articles on mental health-related issues of older people (other than dementia and cognitive disorders) in the IJP till the year 2009. We categorized them into research articles describing psychiatric morbidity of older people, articles specifically looking at depression, late onset psychosis, other mental health issues, case reports and finally editorials/presidential addresses. There were 13 articles describing the nature and prevalence of psychiatric morbidity in late life.[35–47] Most of them were hospital-based studies. According to these reports, people above the age of 60 years constitute 5% of all patients seeking psychiatric help in tertiary care and general hospital settings. Please see Table 2 for an overview of these studies. Five studies looked at the prevalence of mental health morbidity in community samples.[36,38,39,43] Table 3 gives an overview of these studies. The reported prevalence of geriatric psychiatric morbidity in the community varied from 8.9-61.2%. The diagnostic criteria varied across these studies. Some studies looked at the prevalence in people over the age of 50 years while others studied people above 60 years of age. Depression was the commonest psychiatric morbidity. Many studies looked at psychosocial factors associated with depression in late life.[48–55] Variables like female sex, widowed status, nuclear family[48] and stressful life events[51,55] were found to be associated with late life depression. Two studies examined cases of late onset depression, defined as depression having onset after the age of 50 years[51] and after the age of 60 years.[37] Individuals with late onset depression had less hypochondriacal preoccupations and distortion of perception of time than early onset cases.[37] The authors felt that the late onset depression was rather ‘bland’ in its symptom profile when compared to depression with earlier onset.

**Table 2:** Psychiatric morbidity in late life: Hospital-based studies (IJP: 1958-2009)

Study (location)	Setting	Subjects	Diagnosis	Remarks
Venkoba Rao *et al*. (1972) (Madurai, Tamil Nadu)	Tertiary care	97 patients with onset of illness after 50 years of age	Clinical	Subjects: 5% of all patients. Psycho-organic syndromes: 40%
Venkoba Rao A (1981) (Madurai, Tamil Nadu)	Tertiary care, Geropsychiatric clinic	227 patients above 60 years	Clinical	Describes diagnosis and symptoms of depression
Bhogale and Sudarshan (1993) (Belgaum, Karnataka	Tertiary care	238 outpatients and inpatients above 60 years	ICD-9	Analysis of case records. Describes nature and prevalence
Prasad *et al*. (1996) (Bengaluru, Karnataka)	Tertiary care	265 outpatients above 60 years	ICD-9	Analysis of case records. Subjects: 4.17% of outpatients
Pereira *et al*. (2002) (Goa)	Tertiary care	698 outpatients above 60 years	ICD-10	Subjects: 5.4% of outpatients. Affective disorders common: 43.7%
Sing *et al*. (2004) (Chandigarh)	Tertiary care	181 outpatients above 60 years	ICD-10	Analysis of case records. Mood disorders commonest (48.1%)
Sood *et al*.(2006) (Amritsar, Punjab)	Tertiary care	528 inpatients above 65 years of age from other wards	ICD-10	49% had psychiatric co-morbidity. Depression commonest: 25.9%
Tiple *et al*. (2006) (Varanasi, Uttar Pradesh)	Tertiary care and other	84 psychiatry outpatients and others	DSM IV	Depression among psychiatry out patients: 29.8%

**Table 3:** Psychiatric morbidity in late life: Community prevalence (IJP: 1958-2009)

Study (location)	Setting	Subjects	Diagnosis	Remarks
Ramachandran *et al*. (1979) (Chennai, Tamil Nadu)	Suburban community	Random sample of 406 subjects above 50 years	Clinical	Organic psychosis: 3.2%
				Functional disorders: 33.8%
Ramachandran *et al*. (1981) (Chennai, Tamil Nadu)	Suburban community	Random sample of 161 above 60 years	Clinical	Total Prevalence: 33.7%
				Functional disorders: 27.6%
				Illness was mild in most cases. Higher frequency in those from nuclear families or living alone
Venkoba Rao and Madhavan (1982)	Semi-urban community	686 above 60 years	Clinical	Prevalence: 8.9% prevalence of depression: 5.9%
Nandi *et al*. (1997) (Gambhirgachi and Paharpur villages, West Bengal)	Rural community	183 above 60 years	Operational definition for the study	Psychiatric morbidity: 61.2%
				Depression: 52.2%
Tiwari and Srivastava (1998) (Near Lucknow, UttarPradesh)	Rural community	488 above 60 years	ICD-9	Psychiatric morbidity: 42.2%
				Neurotic depression, MDP-depressed type and anxiety state were common

We could only find two articles on late onset psychotic states. The first one was on late paraphrenia. The authors studied 15 cases of ‘paraphrenia’. They included cases that had onset of delusions and/or hallucinations after the age of 60 years.[56] These patients formed about 4% of cases seen in their geropsychiatric clinic. Hallucinatory experiences were present in all cases. Delusions were seen in all cases except one. Most patients had visual or hearing impairment. Ten patients had hearing impairment in this study. Another study from Bengaluru made a comparison between early and late onset schizophrenia.[57] We could not find any studies on delusional disorder. One study reported high prevalence of smoking and alcohol consumption from Ballabgarh in Haryana.[58]

A study from Chennai[59] addressed the important issue of age ascertainment in geriatric research. They used a short checklist which contained multiple historical and personal events to estimate the age. They then compared it with the reported age. Under-reporting of age was common and inaccuracy was noticed even among literate subjects. A stu dy from Manipal assessed different domains of quality of life of older people using the translated Kannada version of the WHO instrument for assessment of quality of life.[60] Erna M. Hoch described the psychosocial issues involved in the healthcare of older individuals.[61] The article emphasized the role of culture and prevalent traditions in the expression of symptoms. Usefulness of a psychodynamic approach was illustrated by giving detailed case histories as examples. Two case reports were published during the period of review. Both were on rare conditions, namely Charles Bonnet Syndrome[62] and dissociative fugue in the elderly.[63]

There were five editorials on issues related to mental health in late life.[64–68] The first editorial on aging was published in 1958. It referred to the challenges associated with aging in a rapidly changing world.[64] It said “today the challenge of old age is made more serious by the increase in the pace of living and scientific advances”. The subsequent editorials also echoed similar sentiments and pointed out the urgent need for development of services and social support systems.

The Geriatric Psychiatry specialty section of the Indian Psychiatric Society (IPS) conducted focus group discussions as part of a qualitative study[69] to elicit the opinion of experts regarding psychogeriatric research and service development. The group felt that there is an urgent need to raise public awareness about mental health conditions of older people. Clinicians should be trained to detect and manage depression and dementia in primary care. Community-based services need to be developed across the country. The continued advocacy by the IPS was evident in the presidential address.[70]

We did not come across studies examining the effects of interventions for psychiatric morbidity. There were no studies on caregiver needs, burden of care or cost of care. The review did not make a systematic effort to identify studies published in other journals as the focus of this review was on the research published in IJP.[71]

## DISCUSSION

Dementia had not been a frequent topic for publication in IJP. However, this does not reflect the progress made in the field of dementia research. There had been many studies in India and their findings were published in other journals. The past decade witnessed active dementia research and networking of researchers.[19,72] Many important epidemiological studies were done in India.[73–80] Both rural and urban populations were studied. A detailed review of these studies appears in the article by Prince MJ in the dementia supplement.[19] The reported prevalence of dementia in the community varied between 0.9-7.5% among the people above 65 years. Methodological issues and the use of different diagnostic criteria could explain the variability in the reported prevalence rates. A simple case-finding method was developed by us at Thrissur.[81] Usefulness of a community-based intervention was reported following a randomized control trial at Goa.[82] These studies, along with studies from other developing countries, form part of the evidence base for the development of the WHO package for management of dementia in low and middle income countries.

Psychiatric morbidity in late life, especially depression, generated lot of research interest in the late seventies and early eighties. Researchers from Madurai and Chennai published many research reports during this period. Studies have shown that 5% of people seeking help in a tertiary care or general hospital setting happen to be older than 60 years. Depression was the commonest disorder and was associated with other physical illnesses. We need more information on the incidence and prevalence of depression from large community samples. A recent study using Geriatric Depression Scale[83] reported a prevalence of 45.9%. Similar rates were reported from West Bengal[43] and Uttar Pradesh.[44] A study from a rural community near Vellore in Tamil Nadu[84] reported a prevalence of 12.7% for depression during the month preceding assessment. They used Geriatric Mental State[85] for evaluation and found geriatric depression to be associated with low income, history of cardiac illnesses, transient ischemic attack, past head injury and diabetes. Having more confidants was a significant protective factor. We need to examine these associations in larger cohorts.

Biological and psychosocial factors could contribute to the development of depression in late life. It is possible to modify many of these factors. Vascular risk factor reduction and adoption of lifestyle changes may help to delay the onset of late life depression and dementia. The usefulness of simple community-based psychosocial interventions for conditions like depression in older people needs to be addressed by future studies.

Development of services for older people with mental health problems will remain a huge public health challenge. Service development in resource-limited settings is not an easy task.[86]

Caregiver support is important in the management of late life mental health problems. Management of disabled older people with behavioral disturbance can be very stressful for the families. Many studies from India had highlighted the importance of identifying and managing behavioral symptoms of dementia.[5–7,87–89] Packages for care for dementia in low and middle income countries had been proposed[90] and management of behavioral symptoms and the provision for caregiver support are given importance in this. Care can be delivered by trained primary care teams, with a paradigm shift towards chronic continuing care and community outreach. Care delivery will be more efficient when integrated with that of other chronic diseases, and more broadly based community support programs for the elderly and disabled. To be successful, all efforts in psychogeriatric service development need to be supported by a clearly spelt out policy on long-term care and political commitment.
